# Expression of *AKR1C3* and *CNN3* as markers for detection of lymph node metastases in colorectal cancer

**DOI:** 10.1007/s10238-014-0298-1

**Published:** 2014-06-17

**Authors:** Chiaki Nakarai, Kayo Osawa, Minami Akiyama, Nagahide Matsubara, Hiroki Ikeuchi, Tomoki Yamano, Seiichi Hirota, Naohiro Tomita, Makoto Usami, Yoshiaki Kido

**Affiliations:** 1 Department of Biophysics, Kobe University Graduate School of Health Sciences, 7-10-2, Tomogaoka, Suma-ku, Kobe, 654-0142 Japan; 2 Department of Surgery, Hyogo College of Medicine, Nishinomiya, Japan; 3 Department of Surgical Pathology, Hyogo College of Medicine, Nishinomiya, Japan; 4 Division of Diabetes and Endocrinology, Department of Internal Medicine, Kobe University Graduate School of Medicine, Kobe, Japan

**Keywords:** *AKR1C3*, *CNN3*, Lymph node metastasis, Colorectal cancer, Real-time quantitative PCR

## Abstract

The aim of the study was to identify a set of discriminating genes that could be used for the prediction of Lymph node (LN) metastasis in human colorectal cancer (CRC), and for this, we compared the whole genome profiles of two CRC cell lines (the primary cell line SW480 and its LN metastatic variant, SW620) and identified eight genes [S100 calcium-binding protein P; aldo–keto reductase family 1(AKR1), member B1 (aldose reductase; *AKR1B1*); AKR1, member C3 (*AKR1C3*); calponin 3, acidic; metastasis associated in colon cancer 1; hemoglobin, epsilon 1; trefoil factor 3; and FGGY carbohydrate kinase domain containing]. These genes were examined by quantitative RT-PCR in tissues and LNs in 14 CRC patients and 11 control patients. The level of *AKR1C3* mRNA expression was significantly different between the Dukes’ stage A, B, and C groups and the control group (*p* < 0.05, *p* < 0.001, and *p* < 0.001) and was also significantly different between Dukes’ stage C and A or B groups (*p* < 0.05 and *p* < 0.001, respectively). The expression of *CNN3* was significantly different between the Dukes’ stage C and B or control groups (*p* < 0.001 and *p* < 0.01, respectively). There were significant correlations between the expression levels of *AKR1C3* and *CNN3*. *AKR1C3* and *CNN3* expressions are more accurate and suitable markers for the diagnosis of LN metastasis than the other six genes examined in this study.

## Background

Lymph node (LN) evaluation is an important factor for determining prognosis in colorectal cancer (CRC). LN metastases cause recurrence of CRC and are related to prognosis and survival [1]. Carcinoembryonic antigen (CEA) was first described as a gastrointestinal oncofetal antigen and is now known to be overexpressed in most carcinomas [2]. CEA is generally used for the detection of LN metastases in CRC [3, 4]. Detection of cytokeratin-20 by RT-PCR in peritumoral, histopathologic, tumor-free LNs is an independent prognostic factor for overall survival in CRC [5]. A biomarker for identifying patients at high risk of metastasis could have extensively clinical applications. Recent evidence indicates that CXC Chemokine Ligand 10 (CXCL10), an interferon-inducible protein, is downregulated in recurrent CRC. Detection of CXCL10 as a prognostic marker for advanced stage CRC patients may help predict clinical outcomes [6]. Our recent study suggests that the expression of the E74-like factor 3 (*ELF3*) gene in LNs signals the possibility of metastases and that *ELF3* may be more suitable than *CEA* as a gene marker for the detection of LN metastases from CRC [7].

In the present study, we compared the whole genome profiles of two isogenic CRC cell lines (the primary cell line SW480 and its LN metastatic variant, SW620) to identify a set of discriminating genes that could be used for the prediction of metastasis in human CRC. A total of 54,359 genes in SW480 and SW620 cells were analyzed using the Whole Genome Bioarray. As a result, we identified 8 genes that had a fivefold increase in the intensity ratio in SW620 cells as compared with SW480 cells and examined by quantitative RT-PCR (qRT-PCR) in tissues and LNs in 14 CRC patients and 11 control patients. The genes selected for examination were S100 calcium-binding protein P (*S100P*); aldo–keto reductase family 1 (AKR1), member B1 (aldose reductase; *AKR1B1*); AKR1, member C3 (*AKR1C3*); calponin 3, acidic (*CNN3*); metastasis associated in colon cancer 1 (*MACC1*); hemoglobin, epsilon 1 (*HBE1*); trefoil factor 3 (intestinal; *TFF3*); and FGGY carbohydrate kinase domain containing (*FGGY*). *S100P* is known to regulate the cellular processes, such as cell cycle progression and differentiation [8, 9]. The protein encoded by *AKR1B1* catalyzes the reduction of a number of aldehydes, including the aldehyde form of glucose, and the protein encoded by *AKR1C3* catalyzes the conversion of aldehydes and ketones [10, 11]. The protein encoded by *CNN3* regulates actin cytoskeleton rearrangement, which is needed for the plasma trophoblast membranes to become fusion competent [12]. *MACC1* is more frequently expressed in advanced CRC [13]. *HBE1* is normally expressed in adult hemoglobin and the leading known cause of a β-thalassemia with gene mutation in Southeast Asia [14]. *TFF3* is expressed in goblet cells in the intestines and the colon, and overexpression of *TFF3* after chemoradiotherapy for rectal cancer is associated with a higher risk of relapse [15]. *FGGY* encodes a member of the *FGGY* kinase family that acts as a phosphotransferase [16]. In this study, we investigated whether these genes could be used as biomarkers for detecting LN metastases of CRC by qRT-PCR.

## Materials and methods

### Microarray analyses

A total of 54,359 genes in two isogenic CRC cell lines (the primary cell line SW480 and its LN metastatic variant, SW620) were analyzed using a CodeLink™ Human Whole Genome Bioarray (Applied Microarrays, Inc. Tempe, AZ, USA). We entrusted microarray analyses to Filgen, Inc. (Nagoya, Japan). The procedure was identical to that of a previous study [17]. Thirty-five genes with a fivefold increase in the intensity ratio in SW620 cells compared with SW480 were arbitrarily defined as being overexpressed in SW620 cells (data not shown). Of the 35 genes that were overexpressed, we selected eight genes (*S100P*, *AKR1C3*, *CNN3*, *AKR1B1*, *MACC1*, *HBE1*, *TFF3*, and *FGGY*) that were extremely overexpressed and were unlikely to be related to inflammation in Table 1.

**Table 1:** Genes upregulated in SW620 cells compared with SW480 cells

Accession number	Gene symbol	Gene name	Intensity ratio
NM_005980	S100P	S100 calcium-binding protein P	48.41
NM_003739	AKR1C3	Aldo–keto reductase family 1, member C3 (3-alpha hydroxysteroid dehydrogenase, type II)	18.19
NM_001839	CNN3	Calponin 3, acidic calmodulin-binding troponin-like protein	16.39
NM_001628	AKR1B1	Aldo–keto reductase family 1, member B1 (aldose reductase)	15.24
NM_182762	MACC1	Metastasis associated in colon cancer 1	11.29
NM_005330	HBE1	Hemoglobin, epsilon 1	9.79
NM_003226	TFF3	Trefoil factor 3 (intestinal)	5.90
NM_018291	FGGY	FGGY carbohydrate kinase domain containing	5.02

### Patients

Twenty-seven tissue specimens (14 tumor specimens and 13 non-tumor specimens) and 125 LNs were dissected from 14 patients with CRC. Non-tumor specimens were located far from primary cancer and confirmed not including tumor cells pathologically. Eleven inflammatory tissue specimens and 35 LNs were dissected as controls from 11 patients who were undergoing surgery for ulcerative colitis (UC). LNs and tissue specimens were obtained from surgical resections performed in the Department of Surgery, Hyogo College of Medicine, Nishinomiya, Japan, between September 2009 and March 2010. The study design was approved by the Ethics Review Committee on Genetic and Genomic Research, Kobe University Graduate School of Health Sciences, Kobe, Japan. Sections of formalin-fixed, paraffin-embedded LNs were examined using HES in the Department of Surgical Pathology, Hyogo Collage of Medicine. All LNs from CRC patients were categorized according to Dukes’ staging system [18] (Table 2). The patients were categorized into three groups: A (*n* = 2), B (*n* = 4), and C (*n* = 8). Almost all cases had lymphatic invasion and/or venous invasion regardless of LN metastasis. In almost all cases, invasion reached the subserosa. Routine hematoxylin-eosin staining (HES) diagnosis of LNs detected metastasis in 4 (28.6 %) out of 14 patients, lymphatic invasion in 10 (71.4 %), and venous invasion in 13 (92.9 %). Extramural cancer deposits (EX) were detected in three cases. EX were defined as cancer foci that were not adjacent to the primary tumor and not associated with LN [19]. Case 10 was EX-positive diagnosed with metastasis-negative LNs on conventional pathologic of staging.

**Table 2:** Clinical and pathological characteristics of patients with colorectal cancer

Case	Dukes’			Histological		
	Location^a^	stage	Histology^b^	Depth	LN metastasis	EX^c^
1	R	A	tub1	sm	–	–
2	R	A	tub1	mp	–	–
3	R	B	tub1	ss	–	–
4	D	B	tub2	ss	–	–
5	A	B	por1	ss	–	–
6	A	B	tub1	ss	–	–
7	A	B	por1	ss	–	–
8	S	B	tub2	ss	–	–
9	A	B	tub2	ss	–	–
10	A	B	muc	se	–	+
11	D	C	por	ss	+	+
12	D	C	tub2	ss	+	–
13	Rb	C	tub2	mp	+	–
14	R	C	tub2	ss	+	+

^a^ *D* descending colon, *A* ascending colon, *R* rectum, *Rb* rectum below peritoneal reflection, *S* sigmoid colon, *T* transverse colon

^b^ *tub1* well-differentiated tubular adenocarcinoma, *tub2* moderately differentiated tubular adenocarcinoma, *por1* poorly differentiated solid adenocarcinoma, *muc* mucinous adenocarcinoma, *sm* submucosa, *mp* muscularis propria, *ss* subserosa, *se* serosa-exposed

^c^ *EX* extramural cancer deposits without lymph node structure

### Tissue preparation/RNA extraction and cDNA synthesis

Tissue preparation, RNA extraction, and cDNA synthesis performed in the same way as described in the previous report [7]. Each RNA from the tissues and LNs was standardized equal concentration.

### Real-time qRT-PCR

One microliter of cDNA was used as the template in the reaction mixture for real-time qRT-PCR. For determination of specific gene expression, each primer was designed with *Perfect real-time primer* (Takara, Ohtsu, Japan). The primers for *S100P* (GenBank Acc. No. NM_005980), *AKR1C3* (GenBank Acc. No. NM_003739), *CNN3* (GenBank Acc. No. NM_001839), *AKR1B1* (GenBank Acc. No. NM_001628), *MACC1* (GenBank Acc. No. NM_182762), *HBE1* (GenBank Acc. No. NM_005330), *TFF3* (GenBank Acc. No. NM_003226), *FGGY* (GenBank Acc. No. NM_018291), and β-actin (*ACTB*; GenBank Acc. No. NM_001101) are listed in Tables 1 and 3. The parameter threshold cycle (Ct) was used as the cycle number to detect the fluorescence increasing. The housekeeping gene *ACTB* was used to calculate the relative level of expression for each gene and data normalization to correct RNA quality and quantity using the 2−ΔΔCt method. qRT-PCR was performed on a MyiQ Real-time PCR System (Bio-Rad, Hercules, CA, USA) using SsoFast EvaGreen Supermix (Bio-Rad, Hercules, CA, USA) according to the manufacturer’s recommendations. The protocol was as follows: initial denaturation at 95 °C for 30 s, followed by 40 cycles of denaturation at 95 °C for 5 s, annealing at the temperature suitable for each gene marker for 10 or 20 s, and extension at 72 °C for 10 s (Table 3). Each sample was assayed in duplicate. A control and two references were included in every run to confirm each examination.

**Table 3:** Primer sequences and PCR conditions used for real-time quantitative RT-PCR

Primer		Sequences	Length	Annealing
S100P	F	5′–TAGCACCATGACGGAACTAGAGACA–3′	182	53 °C 10 s
	R	5′–TGAGCAATTTATCCACGGCATC–3′		
AKR1C3	F	5′–GGATTTGGCACCTATGCACCTC–3′	91	52 °C 10 s
	R	5′–CTATATGGCGGAACCCAGCTTCTA–3′		
CNN3	F	5′–TTCCATACAACCATTGACATTGGAG–3′	127	52 °C 20 s
	R	5′–GGCTGGCACATTTGTTGGTTC–3′		
AKR1B1	F	5′–TATTCACTGGCCGACTGGCTTTA–3′	71	60 °C 10 s
	R	5′–GAACCACATTGCCCGACTCA–3′		
MACC1	F	5′–AGGTCAGCATTGGTTTCACTAGGAG–3′	65	52 °C 20 s
	R	5′–CAATGAGACTGGAGCATGTTTGG–3′		
HBE1	F	5′–CTGAGTGAGCTGCACTGTGACAAG–3′	75	60 °C 10 s
	R	5′–AATCACCATCACGTTACCCAGGA–3′		
TFF3	F	5′–CTGCTGCTTTGACTCCAGGAT–3′	90	63 °C 10 s
	R	5′–CAGCTGGAGGTGCCTCAGAA–3′		
FGGY	F	5′–AGGACCTTGATGATCTTGCCATTC–3′	93	52 °C 20 s
	R	5′–CTGCTGCCTCCATGGCTTCTA–3′		
ACTB	F	5′–TGGCACCCAGCACAATGAA–3′	186	52 °C 10 s
	R	5′–CTAAGTCATAGTCCGCCTAGAAGCA–3′		

### Statistical analysis

Statistical analysis was performed using PASW for Windows version 17.0 (SPSS Japan Inc., Tokyo, Japan). To set cutoff values for each gene marker, receiver operating characteristic (ROC) curve analysis was performed by plotting the true-positive fraction (sensitivity) and false-positive fraction (specificity) pairs with area under the curve (AUC) values for LNs dichotomized according to LN metastasis diagnosed by HES [20, 21]. Data were evaluated using the Kruskal–Wallis test, followed by the Mann–Whitney *U* test with a Bonferroni correction. Analyses of correlations between levels of different mRNA species were performed using a two-tailed Spearman’s rank correlation test. Differences were considered as statistically significant at *p* < 0.05.

## Results

Genes with a fivefold increase in the intensity ratio in SW620 cells as compared with SW480 cells were arbitrarily defined as being overexpressed. Eight candidate genes, *S100P*, *AKR1C3*, *CNN3*, *AKR1B1*, *MACC1*, *HBE1*, *TFF3*, and *FGGY*, were selected on the basis of their remarkable overexpression in SW620 cells and unlikable to be related to inflammation (Table 1). We were examined these genes in tissues and LNs of 14 colorectal cancer patients and 11 controls by real-time qRT-PCR.

**Fig. 1:**
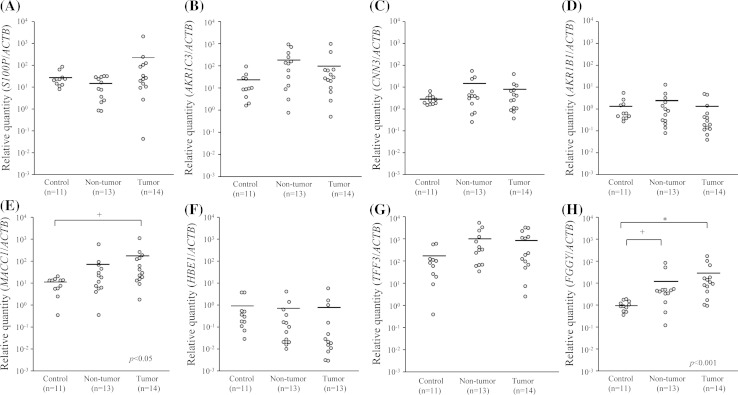
Relative mRNA expression of *S100P*, *AKR1C3*, *CNN3*, *AKR1B1*, *MACC1*, *HBE1*, *TFF3*, and *FGGY* in tissues from colorectal cancer (CRC) patients determined by real-time quantitative RT-PCR. *Dots* showed mRNA levels in 13 non-tumor tissues and 14 tumor tissues from CRC patients compared with 11 inflammatory tissues from ulcerative colitis patients as controls. *Bars* showed means. **a** The relative quantity values (Mean ± SD) of *S100P* are 28.68 ± 24.20, 14.43 ± 12.90, and 199.51 ± 549.78 (control, non-tumor, and tumor). **b** Those of *AKR1C3* are 21.09 ± 26.93, 214.87 ± 291.55, and 124.88 ± 266.74. **c** Those of *CNN3* are 2.86 ± 1.53, 10.44 ± 16.20, and 6.93 ± 10.30. **d** Those of *AKR1B1* are 1.32 ± 1.57, 2.15 ± 3.55, and 0.96 ± 1.63. **e** Those of *MACC1* are 10.04 ± 6.12, 69.13 ± 158.41, and 145.37 ± 289.74. **f** Those of *HBE* are 0.89 ± 1.43, 0.55 ± 1.15, and 0.67 ± 1.61. **g** Those of *TFF3* are 154.79 ± 216.76, 1,116.46 ± 1,610.72, and 908.91 ± 1,158.59. **h** Those of *FGGY* are 1.02 ± 0.50, 13.30 ± 25.12, and 30.65 ± 49.14, respectively. The *p* values are based on Kruskal–Wallis test. +*p*<0.05 and *p < 0.01 are based on Mann–Whitney *U* test with a Bonferroni correction

qRT-PCR was performed to quantify these genes in tumor tissues (*n* = 14), non-tumor tissues from CRC patients (*n* = 13), and inflammatory tissues from patients with UC; the latter tissues served as the controls (*n* = 11). The results are shown in Fig. 1. There were no significant differences in the relative levels of mRNA expression for *S100P*, *AKR1C3*, *CNN3*, *AKR1B1*, *HBE1*, and *TFF3* among tumor tissues, non-tumor tissues, and inflammatory tissues. For *MACC1*, there were significant differences in levels of expression between tumor tissues (Mean ± SD, 145.37 ± 289.74), non-tumor tissues (Mean ± SD, 69.13 ± 158.41), and inflammatory tissues (Mean ± SD, 10.04 ± 6.12) (Kruskal–Wallis test; *p* < 0.05). *MACC1* mRNA expression was significantly different between tumor and inflammatory tissues (Mann–Whitney *U* test with a Bonferroni correction; *p* < 0.05). For *FGGY*, there were significant differences in levels of expression between tumor tissues (Mean ± SD, 30.65 ± 49.14), non-tumor tissues (Mean ± SD, 13.30 ± 25.12), and inflammatory tissues (Mean ± SD, 1.02 ± 0.50) (Kruskal–Wallis test; *p* < 0.001). A subsequent Mann–Whitney *U* test with a Bonferroni correction showed that the mean values for *FGGY* expression were significantly different between non-tumor and inflammatory tissues (*p* < 0.05) and between tumor and inflammatory tissues (*p* < 0.01).

To determine the cutoff values for use in qRT-PCR, ROC curve analysis was performed using relative gene expression values from LNs from CRC patients categorized according to the degree of LN metastasis as evaluated by HES. The cutoff values are shown in Table 4. The AUC values were as follows: *CNN3* = 0.951, SE = 0.037, 95 % confidence interval (CI) = 0.000–1.000, *p* = 0.00002; and *AKR1C3* = 0.919, SE = 0.043, 95 % CI = 0.829–1.000, *p* = 0.00008. The cutoff values for *CNN3* and *AKR1C3* were set at 18.31 with 87.5 % sensitivity and 96.6 % specificity rates and 56.74 with 87.5 % sensitivity and 93.2 % specificity rates, respectively. The AUC values of the other six genes were below 0.9.

**Table 4:** Cutoff values based on ROC curves for eight genes to distinguish lymph node metastasis in colorectal cancer patients

Marker	Cutoff value	AUC value	SE	95 % CI	*p* value
S100P	0.42	0.887	0.036	0.817–0.957	0.00026
AKR1C3	56.74	0.919	0.043	0.829–1.000	0.00008
CNN3	18.31	0.951	0.037	0.000–1.000	0.00002
AKR1B1	1.35	0.755	0.054	0.649–0.862	0.01592
MACC1	4.13	0.774	0.095	0.587–0.960	0.00981
HBE1	0.18	0.833	0.052	0.731–0.936	0.00165
TFF3	1.97	0.713	0.105	0.507–0.918	0.04471
FGGY	5.90	0.626	0.102	0.425–0.827	0.23394

**Fig. 2:**
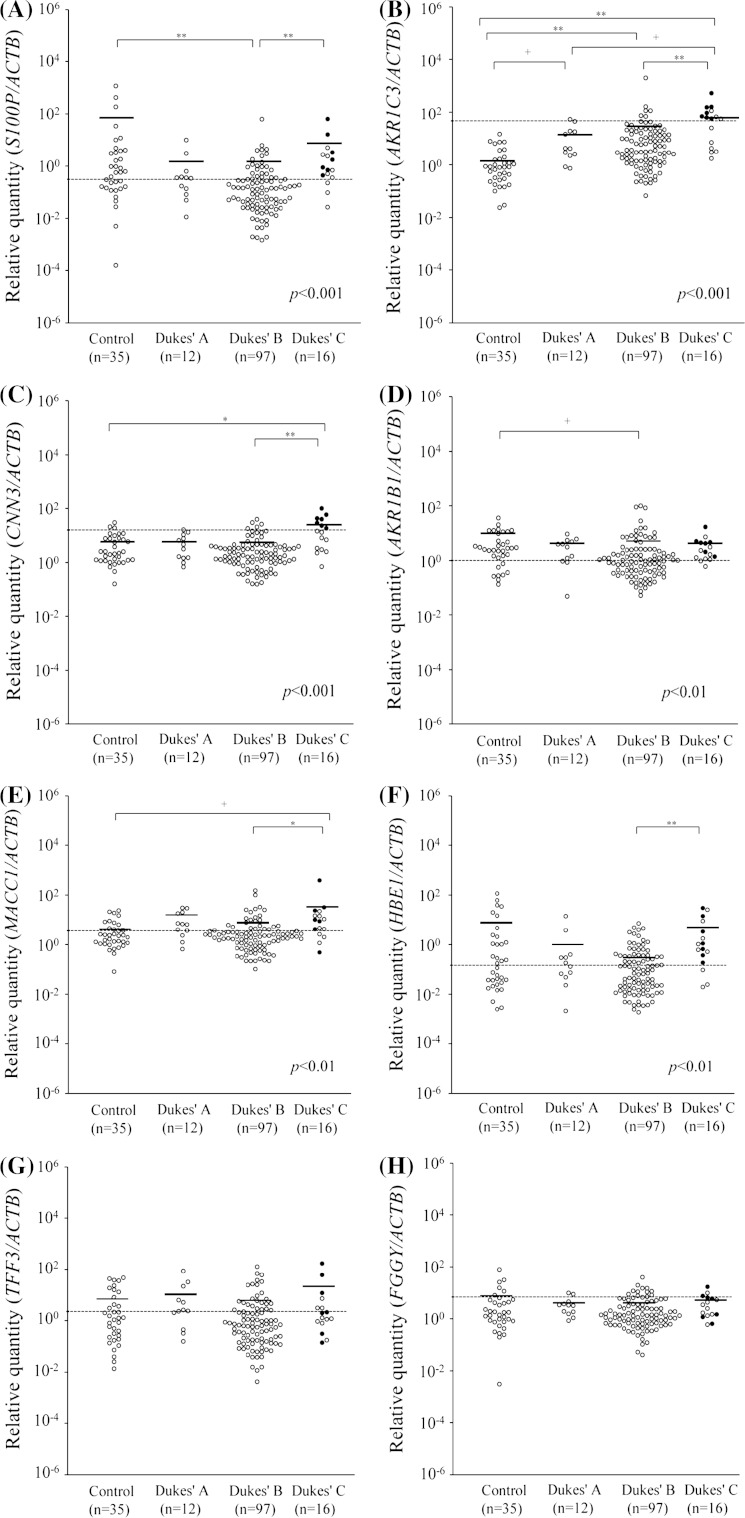
Relative mRNA expression of *S100P*, *AKR1C3*, *CNN3*, *AKR1B1*, *MACC1*, *HBE1*, *TFF3*, and *FGGY* in lymph nodes (LNs) from colorectal cancer (CRC) patients categorized by Duke’s classification. *Dots* showed mRNA levels in 125 LNs from CRC patients with Dukes’ stage A, B, and C, compared with 35 LNs from ulcerative colitis patients as controls. *Black dots* indicate LNs with tumor cells, and *gray dots* indicate LNs without tumor cells identified by hematoxylin–eosin staining. *Broken lines* show cutoff values of eight genes in Table 4. *Bars* showed means. **a** The relative quantity values (Mean ± SD) of *S100P* are 53.17 ± 208.51, 1.30 ± 2.86, 1.10 ± 6.44, and 6.24 ± 16.00 (control, Dukes’ A, Dukes’ B, and Dukes’ C). **b** Those of *AKR1C3* are 1.97 ± 3.14, 13.99 ± 17.82, 34.03 ± 201.50, and 87.72 ± 127.72. **c** Those of *CNN3* are 5.46 ± 6.73, 5.73 ± 5.28, 4.06 ± 6.39, and 22.97 ± 26.78. **d** Those of *AKR1B1* are 5.49 ± 7.29, 3.44 ± 2.72, 5.15 ± 15.99, and 3.64 ± 4.01. **e** Those of *MACC1* are 4.53 ± 6.06, 10.87 ± 10.81, 6.43 ± 18.75, and 34.17 ± 95.53. **f** Those of *HBE* are 8.57 ± 22.92, 1.61 ± 4.06, 0.48 ± 1.14, and 5.52 ± 9.51. **g** Those of *TFF3* are 7.75 ± 13.53, 13.54 ± 25.01, 5.48 ± 16.74, and 16.70 ± 43.65. **h** Those of *FGGY* are 6.45 ± 14.38, 3.67 ± 2.87, 3.20 ± 5.24, and 4.41 ± 4.33, respectively. The *p* values are based on Kruskal–Wallis test. +*p*<0.05, *p < 0.01 and **p < 0.001 are based on Mann–Whitney *U* test with a Bonferroni correction

To investigate whether each gene was overexpressed in metastatic LNs from CRC, we measured mRNA expression in 12 LNs from patients categorized into Dukes’ stage A, 97 LNs from patients categorized into Dukes’ stage B, and 16 LNs from Dukes’ stage C. As a control, we also measured mRNA expression in 35 LNs dissected from UC patients. As shown in Fig. 2, each level of *S100P*, *AKR1C3*, *CNN3,* *AKR1B1*, *MACC1*, and *HBE1* mRNA expression was significantly different among the Dukes’ stage A, B, and C groups and the control group (*S100P*, *AKR1C3*, *CNN3*: *p* < 0.001,; *AKR1B1*, *MACC1,* *HBE1: p* < 0.01, respectively; Kruskal–Wallis test). The level of *AKR1C3* mRNA expression was significantly different between the Dukes’ stage A, B, and C groups and the control group (*p* < 0.05, *p* < 0.001, and *p* < 0.001, respectively; Mann–Whitney *U* test with a Bonferroni correction) and was also significantly different between Dukes’ stage C and Dukes’ stage A or Dukes’ stage B groups (*p* < 0.05 and *p* < 0.001, respectively). A subsequent Mann–Whitney *U* test with a Bonferroni correction showed that expression of *CNN3* and *MACC1* was significantly different between the Dukes’ stage B and C groups (*p* < 0.001, *p* < 0.01, respectively) and between the control and the Dukes’ stage C groups (*p* < 0.01, *p* < 0.05, respectively). The *S100P* and *HBE1* were significantly different between the Dukes’ stage B and C groups (*p* < 0.001, *p* < 0.001, respectively), and the *S100P* and *AKR1B1* were significantly different between the control and the Dukes’ stage B groups (*p* < 0.001, *p* < 0.05, respectively). On the other hand, there were no significant differences in *TFF3* and *FGGY* mRNA expression among those four groups. The mRNA expression of *AKR1C3*, *CNN3*, and *MACC1* was significantly higher in the Dukes’ stage C group than in the control group.

Furthermore, to investigate the correlation between the mRNA levels for the eight biomarkers, we compared mRNA expression in the LNs of CRC patients and controls. LNs from the controls and each staging group were analyzed separately. The results worthy of special mention are shown in Table 5. There were significant correlations between the levels of *AKR1C3* and *CNN3* mRNA expression overall (*r* = 0.635; *p* < 0.001), in Dukes’ A (*r* = 0.888; *p* < 0.001), Dukes’ B (*r* = 0.712; *p* < 0.001), and Dukes’ C (*r* = 0.844; *p* < 0.001) groups and in the control group (*r* = 0.475; *p* < 0.01).

**Table 5:** Correlation between expression levels of biomarker *AKR1C3* and *CNN3* mRNAs in lymph nodes of colorectal cancer patients and controls

Compared marker	All lymph nodes		Control		Dukes’ A		Dukes’ B		Dukes’ C	
	*r* ^a^	*p* value^a^	*r*	*p* value	*r*	*p* value	*r*	*p* value	*r*	*p* value
AKR1C3 vs CNN3	0.635	<0.001	0.475	<0.01	0.888	<0.001	0.712	<0.001	0.844	<0.001

^a^ *r* and *p* values obtained using two-tailed Spearman’s rank correlation test

The relationships between the qRT-PCR results and histological examination are shown in Table 6. The results can be summarized as follows: There were 7 of 7 true-positives for *S100P*, *AKR1C3*, *CNN3*, *AKR1B1*, and *HBE1*; 6 of 7 for *MACC1*; 5 of 7 for *TFF3*; and 3 of 7 for *FGGY* (statistical analysis was omitted due to low case numbers).

**Table 6:** Lymph node (LN) metastases detected by real-time quantitative RT-PCR (qRT-PCR) and histological examination

Marker	Histological LN metastasis	qRT-PCR	
		Positive^a^	Negative
		*n* (%)	*n* (%)
S100P	Positive	7/7 (100)	0/7 (0)
	Negative	29/118 (24.6)	89/118 (75.4)
AKR1C3	Positive	7/7 (100)	0/7 (0)
	Negative	8/118 (6.8)	110/118 (93.2)
CNN3	Positive	7/7 (100)	0/7 (0)
	Negative	4/118 (3.4)	114/118 (96.6)
AKR1B1	Positive	7/7 (100)	0/7 (0)
	Negative	49/118 (41.5)	69/118 (58.5)
MACC1	Positive	6/7 (85.7)	1/7 (14.3)
	Negative	36/118 (30.5)	82/118 (69.5)
HBE1	Positive	7/7 (100)	0/7 (0)
	Negative	46/118 (39.0)	72/118 (61.0)
TFF3	Positive	5/7 (71.4)	2/7 (28.6)
	Negative	40/118 (33.9)	78/118 (66.1)
FGGY	Positive	3/7 (42.9)	4/7 (57.1)
	Negative	16/118 (13.6)	102/118 (86.4)

^a^Cutoff values as indicated in Table 4

## Discussion

Recently, there have been many reports regarding the use of novel gene markers to detect colon tumors in early stages and diagnose the status of the disease appropriately. The purpose of this study was to find new markers for the detection of LN metastases in CRC by qRT-PCR. On the basis of comparative microarray analyses, we identified eight candidate genes (*S100P*, *AKR1C3*, *CNN3*, *AKR1B1*, *MACC1*, *HBE1*, *TFF3*, and *FGGY*).

In this study, *S100P* was remarkably overexpressed in SW620 cells as compared with SW480 (intensity ratio = 48.41). It has been reported that expression of the *S100P* mRNA and protein is significantly higher in cancerous regions than in non-cancerous tissues [22]. It has been known to express in cancer cells in adult specifically and to mediate tumor growth, drug resistance, and metastasis [23–25]. However, our study revealed that there are no significant differences in levels of *S100P* expression between non-tumor tissues and tumor tissues. Our findings conflict with those reported by others.

As a result of our ROC curve analysis, we conclude that *AKR1C3* and *CNN3* expression are more accurate and suitable for the diagnosis of LN metastasis than the other 6 genes. The AUC values of *AKR1C3* and *CNN3* were 0.919 and 0.951, respectively, whereas our previous data showed AUC values for *ELF3* and *CEA* of 0.955 and 0.903, respectively [7]. From this point of view, *AKR1C3* and *CNN3* are more accurate markers than *CEA* and may be considered to be as accurate as *ELF3*.

The mRNA expressions of *AKR1C3* and *CNN3* were found to be significantly higher in the Dukes’ stage C group than in the control groups. The *AKR1C3* mRNA expression was also found to be significantly different between the Dukes’ stage C group and the other Dukes’ groups. To our knowledge, this report is the first study on LN metastasis in CRC that has focused on *AKR1C3* and *CNN3*. We found that the mRNA expression of both genes in primary tumor tissues was different from that in non-tumor or inflammatory tissues. In addition, there was a significant correlation between *AKR1C3* and *CNN3* mRNA expression (Table 5). *AKR1C3* expression has been demonstrated in sex hormone-dependent tissues, including breast [11], endometrial [26], testis [27], and prostate tissues [11] as well as in sex hormone-independent tissues, including kidney, bladder, and urothelial tissues [28]. Elevated expression of *AKR1C3* has been identified in prostate and breast cancer and is correlated with the aggressiveness of the disease [11, 29, 30]. Positive immunoreactivity *AKR1C3* was widely present in both adenocarcinoma and squamous cell carcinoma of the lung and gastroesophageal junction [31]. A previous study showed that *AKR1C3* mRNA and protein were overexpressed in castration-resistant prostate cancer tissue as compared to benign prostate and primary prostate cancer tissue [32]. CNN3 was identified the gene in tumorigenic parameter as ovarian cancer and mucosa-associated lymphoid tissue lymphoma [33, 34].

*MACC1* is a key regulator of the hepatocyte growth factor receptor pathway, including in cellular growth, invasiveness, and metastasis, and is useful to identify the poor prognosis in CRC patients [35]. We found that the level of *MACC1* mRNA expression differs between primary tumor tissues and inflammatory tissues (*p* < 0.05, Kruskal–Wallis test). As *MACC1* overexpression was not found in all the histologically positive LNs examined in this study, we conclude that *MACC1* may be inferior to *AKR1C3* and *CNN3* in detecting LN metastases.

However, *AKR1B1*, *HBE1*, *TFF3*, and *FGGY* were not suitable for detecting LN metastases in view of the fact that there were no significant differences in the expression levels of these genes between the control group and the Dukes’ stage C group. *AKR1B1* is overexpressed in human tumors, such as those found in liver, breast, and lung cancer, and may play a important role in the development and progression of cancer [36]. There is no previous report of *HBE1*’s expression in cancer cells. *TFF3* expression may play a role in promoting LN metastases in CRC [37]. *FGGY* expression has been recently associated with an increased susceptibility to sporadic amyotrophic lateral sclerosis [38]. We would like to emphasize our study limitations, especially the number of patients were not enough for definitive conclusion. Thus, it may be biased by the relatively small number of patients.

In conclusion, *AKR1C3* and *CNN3* expression are more accurate and suitable markers for the diagnosis of LN metastasis than the other six genes examined in this study. We found that the difference in *AKR1C3* expression between all Dukes’ stage groups and the control group was statistically significant. In addition, there were significant correlations between the expression levels of *AKR1C3* and *CNN3*. *AKR1C3* and *CNN3* might be more suitable than the other six genes as gene markers for the detection of LN metastases from CRC and require further verification as biomarkers in a larger population study.

## Abbreviations

LN: Lymph node
CRC: Colorectal cancer
UC: Ulcerative colitis
CEA: Carcinoembryonic antigen
CXCL10: CXC chemokine ligand 10
ELF3: E74-like factor 3
S100P: S100 calcium-binding protein P
AKR1: Aldo–keto reductase family 1
AKR1B1: AKR1, member B1
AKR1C3: AKR1, member C3
CNN3: Calponin 3, acidic
MACC1: Metastasis associated in colon cancer 1
HBE1: Hemoglobin, epsilon 1
TFF3: Trefoil factor 3
FGGY: FGGY carbohydrate kinase domain containing
HES: Hematoxylin–eosin staining
